# A Laparoscopic Management Combined with a Flexible Ureteroscope for Ureteral Polyps of More Than 3 cm Length

**DOI:** 10.1089/cren.2016.0058

**Published:** 2016-06-01

**Authors:** Mitsunori Matsuo, Kousuke Ueda, Kiyoaki Nishihara, Makoto Nakiri, Shunsuke Suyama, Katsuaki Chikui, Shuichiro Hayashi, Hirofumi Kurose, Naoyuki Ogasawara, Shigetaka Suekane, Tsukasa Igawa

**Affiliations:** Department of Urology, Kurume University School of Medicine, Kurume, Japan.

## Abstract

Ureteral polyps are benign tumors of the ureter, which are relatively rare. The etiology has proposed various hypotheses, involving chronic inflammation and congenital disease. Most of them are commonly diagnosed in the upper ureter including the ureteropelvic junction. Some studies have reported polypectomy using a holmium laser, but several studies presented laparoscopic ureteroureterostomy for patients in whom the mentioned procedure is difficult. We underwent laparoscopic ureteroureterostomy with a combination of flexible ureteroscope for ureteral polyps of more than 3 cm length. We used ureteroscopy with a laparoscopic approach to minimize the length of ureter resection. Using the light guide of ureteroscopy is useful to decide the exact and minimal excision range for ureteroureterostomy.

## Introduction

Ureteral polyps are benign tumors of the ureter, which are relatively rare. Although the etiology remains to be clarified, various hypotheses, involving chronic inflammation and congenital disease, have been proposed. Many patients show hydronephrosis regardless of the tumor site. It causes flank pain or hematuria in some patients. Concerning treatment, transurethral resection using a holmium laser was recently reported, but several studies presented laparoscopic ureteroureterostomy for patients in whom the mentioned procedure is difficult. In this study, we report a patient with multiple ureteral polyps, measuring more than 3 cm in length, in whom laparoscopic ureteroureterostomy using a flexible ureteroscope was performed, leading to favorable results, because transurethral laser resection was difficult.

## Case Report

The patient was a 36-year-old male. Left lumbar pain appeared, and he consulted a local clinic. Abdominal ultrasonography showed left hydronephrosis. For detailed examination and treatment, he was referred to our department. Contrast-enhanced CT revealed left hydronephrosis and a tumorous lesion of the upper ureter, with enhancement effects. Under a tentative diagnosis of left ureteral cancer, transurethral retrograde ureteroscopy and tumor biopsy were performed. Endoscopically, a papillary tumor involved the inner cavity of the ureter, and it was difficult to examine the tumor base. Furthermore, there was no influx of contrast medium into an area superior to the tumor. Under a flexible ureteroscope, laser resection was conducted, but it was difficult to resect the tumor. The histopathologic findings suggested fibroepithelial polyps (FEPs).

**FIG. 1.**
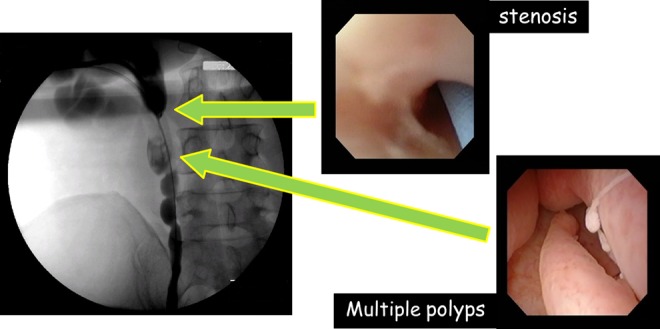
Anterograde pyelography shows a severe ureteral stenosis and continuously about 4 cm of filling defect on the distal side (*left*). Close-up view of the stenosis site and ureteral polyps with flexible ureteroscope (*center*/*right side*).

**FIG. 2.**
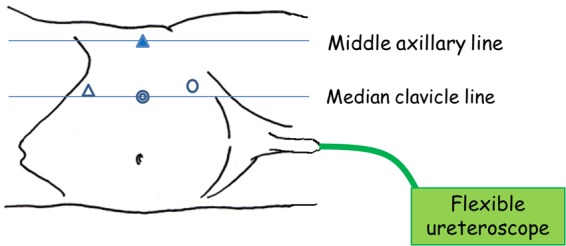
It shows positions of the patient and the positions of trocars. 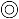: camera port (12 mm), △: left hand for operator (7 mm),○: right hand for operator (7 mm), ▲: 7 mm port for assistant. Flexible ureteroscope was inserted in a right-sided position.

**FIG. 3.**
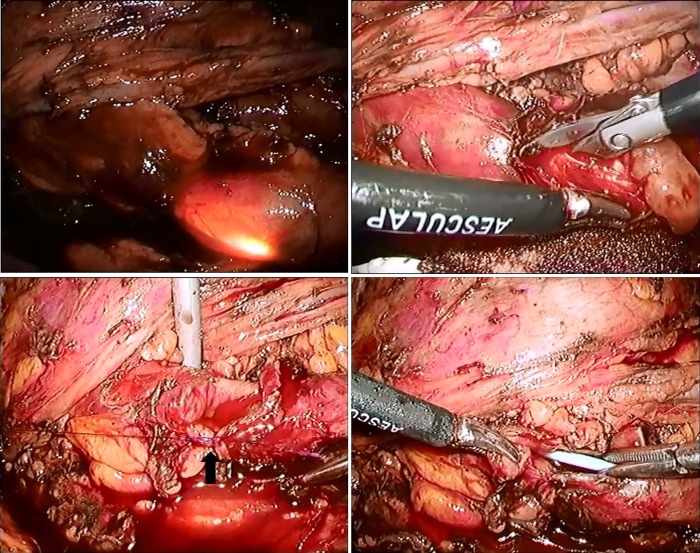
Intraoperative laparoscopic view: The light guide of ureteroscope can confirm from the ureter (*top left*); multiple polyps bulge out through ureteral incision (*top right*); arrow indicates anastomosis on posterior wall of the ureter (*bottom left*); ureteral stent inserted through the urethra easily (*bottom right*).

**FIG. 4.**
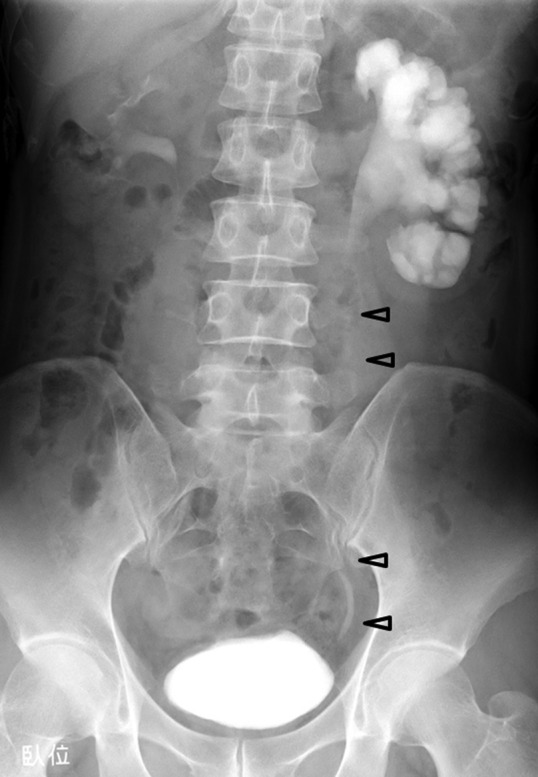
Intravenous urography showing left renal pelvis to lower ureter at 3 months after the operation (*arrow heads*).

As tumor observation was insufficient on retrograde ureteroscopy, percutaneous anterograde ureteroscopy was performed. Under a flexible ureterorenoscope, multiple polyps measuring about 4 cm in length and the ureteral stenosis on the cephalic side were confirmed continuously (Fig. 1). As transurethral treatment was difficult, laparoscopic ureteroureterostomy using a flexible ureteroscope was performed. Surgery was conducted using a transperitoneal approach in a right-sided position. Concerning the position of trocars, ports were established in accordance with standard pyeloplasty. We used 12-mm camera ports and three 7-mm ports for the left and right hands and an assistant (Fig. 2). Although exfoliation around the kidneys and ureter was sufficiently performed, exfoliation on the lateral side of the kidneys was not conducted for suture. The ureter on the distal side was exfoliated until the iliac artery intersection. Subsequently, a flexible ureteroscope was transurethrally inserted to identify ureteral polyps and the site of stenosis. Simultaneously, the site of resection was decided using a laparoscope, regarding the ureteroscope's light guide as a mark (Fig. 3, top left). The length of the ureter to be resected was ∼3.5 cm (Fig. 3, top right). Spatulation (∼5 mm) was performed on the cephalic and leg sides of the ureteral stump so that a 180° contralateral position was established. Using a 4-0 monofilament, knotted suture was conducted (Fig. 3, bottom left). A 6F Double-J stent was transurethrally inserted (Fig. 3, bottom right). After confirming the absence of tension in the ureter after suture, surgery was completed. The operative time was 219 minutes. The volume of blood loss was small. There were no perioperative complications. The final pathologic report revealed FEPs and inflammatory cells infiltration. A Double-J stent was removed 6 weeks after the operation. At 3 months after the procedure, intravenous urography showed no obstruction and the renal pelvis to lower ureter clearly (Fig. 4).

## Discussion

FEPs of the ureter are a relatively rare benign tumor. They are frequently present in 30 and 40 years old males. They usually occur in the left ureter twice more than in the right, and commonly most of them are diagnosed in the upper ureter including the ureteropelvic junction. Adey and colleagues reported that FEPs of the ureter were rare in children. They confirmed only 9 cases of 1710 ureteropelvic junction obstructions on whom pyeloplasty was performed.^1^ Preoperative radiographic diagnosis of the ureteral polyps is difficult that distinguish right from uteretal carcinoma and radiolucent calculi. It is necessary to perform ureteroscopy or transurethral biopsy to confirm them.^2^

To treat FEPs of the ureter, partial ureterectomy under laparotomy or nephrectomy has been performed in ancient times. With subsequent advances in endoscopic devices, transurethral resection has been selected to treat pedunculated polyps. Some studies have reported polypectomy using a holmium laser, which has been routinely used to treat calculi since its appearance. Even if most of FEPs are possible for resection, the base of the polyps may remain without being sufficiently resectable.^3^ Recently, several reports described that laparoscopic pyeloplasty or ureteroureterotomy was performed for large FEPs. Tamasz and colleagues described that laparoscopy being the treatment for FEPs is a useful and minimally invasive technique. When the endoscopic procedure is difficult or impossible in large polyps, a procedure of the surgeon's choice may be considered.^4^ In our department, resection using a holmium laser has also been performed as a first-choice treatment for pedunculated polyps. However, laser resection is not always possible for patients with sessile or multiple polyps. We have rarely encountered patients in whom treatment was difficult. In such cases, it is necessary to perform ureteroureterostomy after partial ureterectomy at the polyp site. When the length of the ureter to be resected is long, end-to-end anastomosis of the ureter is difficult, requiring intestinal utilization in some cases. In the resection of more than 4 cm ureter including the constriction, we predicted difficulty and uncertainty when we identify the excision site or determine the length of the resection in the laparoscopic surgery alone. Therefore, to minimize the extent of the ureter to be resected, we have combined a flexible ureteroscope. It was possible to accurately identify the normal site of the ureter under ureteroscope-guided observation and decide on the extent of resection through the abdominal cavity, regarding its light guide as a mark. As a result, the extent of resection was shorter than estimated on preoperative assessment, and ureteroureterostomy was effectively achieved. Furthermore, transurethral operations were combined, facilitating the insertion of a ureteral stent (Double-J stent) without loading at the anastomotic site. In addition, we minimized perirenal exfoliation, considering that circumferential perirenal exfoliation may cause the kidneys to completely separate, influencing subsequent operations, such as ureterostomy; therefore, operations, such as suture, could be promoted readily. The present case suggests that ureteroureterostomy is possible even when resecting a 4- to 5-cm area of the ureter. Laparoscopic ureteroureterostomy combined with a flexible ureteroscope may become a major treatment option for patients including those in whom transurethral treatment is difficult.

In conclusion, we performed laparoscopic management using a ureteroscope to minimize the excision site. The use of the ureteroscope's light guide as a mark was useful to accurately decide on the extent of the resection.

## Abbreviations Used

FEPs: fibroepithelial polyps
